# Covid-19 in Hospitalized Patients Admitted from Skilled Nursing Facilities: Factors Associated with Mortality

**DOI:** 10.1093/geroni/igab046.3377

**Published:** 2021-12-17

**Authors:** Rongfang Zhan, Cheng Yin, Liam O'Neill

**Affiliations:** 1 University of North Texas, Corinth, Texas, United States; 2 University of North Texas, University of North Texas, Texas, United States

## Abstract

Background: Nursing home residents were impacted disproportionately by the coronavirus because of their vulnerabilities. Although many studies concentrated on risk factors associated with mortality of hospitalized patients, there were limited studies epitomizing them from skilled nursing facilities to hospitals. The study aims to identify inpatients’ characteristics on demographics, hospital admission types, insurance types, and chronic diseases associated with mortality among our cohort patients in Texas. Methods: Individuals above 50 years, diagnosed with Covid-19, and admitted from skilled nursing facilities were included in the retrospective cohort study. Pearson’s Chi-Square and Mann-Whitney tests were applied to measure four major perspectives between survivors and non-survivors. Then, a binary logistic regression was employed to determine the association between independent variables and mortality. Results: A total of 218 patients were included in the study, of which 54 (24.8%) died during hospitalization. According to the univariate analysis, expired patients were more likely to be emergency admission (p = 0.001), elective admission (p = 0.02), Medicaid as primary payment (p = 0.034), heart disease (p = 0.027), CKD (p = 0.03), and hypertension (p = 0.002). The binary logistic regression revealed that hypertension (OR = 3.176, 95% CI: 1.200-8.409, p = 0.02) and Medicaid (OR = 2.637, 95% CI: 1.287-5.405, p = 0.008) as primary payment had significantly high odds of mortality. Conclusion: Hypertension and Medicaid as primary payment are the strongest predictive factors associated with mortality and suggest that hospitals in Texas distribute critical care and resources while prevent and treat them to increase survival rates.

